# State-of-the-Art Knee Exoskeleton for Gait Assistance and Rehabilitation: Systematic Review

**DOI:** 10.2196/94916

**Published:** 2026-08-04

**Authors:** Dilnoza Karibzhanova, Aibek Niyetkaliyev, Amna R Khawaja, Aray Zhaisanbek, Zeeshan, Ardana Utepbergen, Prashant K Jamwal, Kassymbek Ozhikenov, Aiman Ozhikenova, Chingiz Alimbayev, Zhadyra Alimbayeva, Zhanar Bigaliyeva, Lashin Bazarbay, Serik Didar

**Affiliations:** 1 School of Medicine, Nazarbayev University, Kabanbay Batyr 53 Street, Astana, 010000, Kazakhstan, 7 7753575664; 2 School of Engineering and Digital Sciences, Nazarbayev University, Astana, Kazakhstan; 3 Department of Physical Therapy, Rehabilitation Science and Athletic Training, University of Kansas Medical Center, Kansas City, KS, United States; 4 Department of Robotics and Technical Means of Automation, Satbayev University, Almaty, Kazakhstan; 5 Qazaq Youth Science Hub LLP, Almaty, Kazakhstan

**Keywords:** knee exoskeleton, gait rehabilitation, wearable robotics, stroke, spinal cord injury, orthopedic rehabilitation

## Abstract

**Background:**

Knee exoskeletons represent a significant advancement in wearable robotic technology, developed to enhance gait assistance and facilitate rehabilitation processes. These devices are increasingly used in clinical and research environments worldwide. Nonetheless, the existing empirical evidence supporting their biomechanical and functional outcomes lacks both breadth and clarity. Further investigation is essential to elucidate the specific effects of knee exoskeletons on gait dynamics and rehabilitation efficacy.

**Objective:**

This study aims to systematically review the clinical and biomechanical evidence on knee exoskeletons used for gait assistance and rehabilitation.

**Methods:**

A systematic search of databases—PubMed, Web of Science, IEEE Xplore, and Scopus—was conducted to identify eligible studies published between January 2015 and March 2025. Studies were included if they investigated knee exoskeleton systems in human participants for gait assistance and rehabilitation. Randomized controlled trials, observational studies, and experimental investigations were eligible. Exclusion criteria were multijoint exoskeletons, nonhuman studies, and simulation-based analyses. Two reviewers independently extracted study characteristics, participant demographics, device features, intervention protocols, and clinical and biomechanical outcomes. Risk of bias was assessed using RoB-2 (Cochrane Risk of Bias 2) for randomized controlled trials and ROBINS-I (Risk of Bias in Nonrandomized Studies of Interventions) for nonrandomized studies.

**Results:**

Thirty-two studies met the inclusion criteria. Most investigations involved healthy adults in feasibility or pilot settings, while a smaller subset included individuals with stroke, spinal cord injury, or orthopedic conditions or pediatric movement disorders. Across studies, knee exoskeletons demonstrated short-term improvements in spatiotemporal gait parameters, including increased gait speed and step length, improved knee joint kinematics, and reductions in muscular effort or metabolic demand. However, substantial heterogeneity in device design, outcome metrics, and intervention duration limits comparability across studies. Sample sizes were typically small, and follow-up outcomes were rarely reported, restricting the interpretation of the long-term clinical efficacy of these devices.

**Conclusions:**

Knee exoskeletons demonstrate promising short-term functional and biomechanical benefits and are increasingly feasible within structured rehabilitation settings. While current evidence is limited by small sample sizes and brief follow-up periods, emerging data suggest potential for enhancing task-specific gait training. Larger, well-designed trials are needed to determine sustained clinical impact and integration into routine rehabilitation practice.

## Introduction

Gait disorders and knee joint dysfunction are the most common consequences of neurological and orthopedic diseases, including postoperative conditions such as total knee replacement [1]. Neurological gait disorders are commonly associated with stroke, spinal cord injury, cerebral palsy, and degenerative conditions. At the same time, orthopedic pathologies such as osteoarthritis, joint degeneration, and skeletal deformities of the lower extremities also contribute to gait disorders and progressive disability [2,3]. Knee disorders impose a substantial and growing global burden across all populations. Osteoarthritis of the knee is especially prevalent, affecting approximately 4%‐5% of people worldwide (365 million in 2019) [4] and, by some estimates, about 16% of adults aged >40 years (654 million) [5]. Traumatic knee injuries (eg, sports or accident injuries) also contribute significant morbidity, especially in younger and athletic populations, often precipitating earlier osteoarthritis [6]. Even after surgical treatment (eg, total knee arthroplasty), many patients remain disabled: roughly 15%‐30% report persistent pain or functional limitation postoperatively [7,8]. Economic costs are correspondingly high: one systematic review found mean annual costs of €11,100 (€1=US $1.16 as of March 10, 2026) per knee/hip patient with osteoarthritis [9], and national expenditures reflect this trend. For example, osteoarthritis-related costs in Germany rose from €8.6 billion in 2015 to €12.1 billion in 2020 [10]. These impairments significantly affect mobility, functional independence, and quality of life across adult and pediatric populations.

The knee joint plays a central role in shock absorption, load transfer, and movement during walking and functional loads [11]. Several technology-enhanced modalities have been investigated as adjuncts to conventional knee rehabilitation, except for robotic exoskeletons. Virtual reality–based training has demonstrated clinically meaningful improvements in pain, strength, and function. In patients undergoing anterior cruciate ligament reconstruction, a meta-analysis of 9 randomized controlled trials reported significant reductions in pain and improvements in knee function, quadriceps strength, and knee flexion range of motion compared with standard therapy [12]. Similarly, pooled evidence from studies of rehabilitation in total knee arthroplasty (TKA) showed modest but significant improvements in pain, balance, and knee-specific function with virtual reality interventions [13]. Telerehabilitation and app-guided programs have also demonstrated outcomes comparable to face-to-face therapy, including improved pain relief, knee flexion, and quadriceps strength after TKA [14]. Wearable biofeedback systems further support functional recovery, with faster Timed Up and Go performance and higher patient satisfaction reported following TKA rehabilitation [15].

Despite these benefits, most nonrobotic technologies rely on feedback-based strategies and do not provide direct mechanical assistance to the knee joint during functional tasks.

Wearable knee exoskeletons offer a complementary approach by delivering task-specific, assist-as-needed mechanical support during gait, enabling high-repetition training while reducing physical demand and discomfort [16,17]. Exoskeleton-assisted rehabilitation has been associated with improved gait performance, muscle activation, and functional independence in neurological and orthopedic populations [18,19]. Emerging evidence also suggests that robotic gait training may promote central neuroplastic adaptations, supporting recovery beyond peripheral biomechanics [20]. In addition, knee exoskeletons are increasingly explored for fall prevention, with integrated recovery assistance shown to improve swing-limb kinematics and reduce fall risk in mobility-impaired populations [21]. This highlights the growing use of exoskeletons as both therapeutic and protective devices.

A wide range of technology-based interventions, including virtual reality, telerehabilitation, neuromuscular stimulation, and sensor-driven feedback systems, have been implemented in orthopedic and neurological knee rehabilitation [22-26]. Among these approaches, robotic knee exoskeletons have attracted increasing interest due to their capacity to provide biomechanical support and task-specific training. Despite growing evidence of their potential benefits, substantial gaps remain regarding consistency, affordability, and comparative effectiveness across technologies. With a focus on clinical outcomes, implementation challenges, and integration into rehabilitation care pathways, this systematic review examines knee exoskeletons for gait assistance and rehabilitation, with particular attention to their clinical applications and mechanical components.

This systematic review aimed to critically evaluate knee exoskeletons used for gait assistance and rehabilitation. The objectives were to identify the types of knee exoskeleton systems investigated, the clinical conditions and patient populations in which these devices have been applied, and the reported clinical and biomechanical outcomes associated with their use. In addition, the review identifies key gaps in the existing literature and proposes directions for future research to support the safe, effective, and scalable adoption of advanced rehabilitation technologies.

## Methods

### Protocol and Registration

This systematic review was registered on PROSPERO (International Prospective Register of Systematic Reviews) under registration number CRD420261285256. The review was conducted and reported in accordance with the PRISMA (Preferred Reporting Items for Systematic Reviews and Meta-Analyses) 2020 guidelines (Checklist 1). Minor methodological changes were made during full-text screening to align the risk-of-bias assessment tools with the final included study designs. These refinements did not affect the overall objectives, eligibility criteria, or review conclusions.

### Search Strategy

A comprehensive literature search was conducted across 4 electronic databases: PubMed, Scopus, Web of Science, and IEEE Xplore. The search covered studies published between January 2015 and March 2025, with the final search performed in March 2025. These databases were selected to ensure coverage of biomedical, rehabilitation, engineering, robotics, and wearable technology literature.

To improve sensitivity and minimize the risk of missing relevant studies, the search strategy combined keywords and synonyms related to exoskeleton technologies and the knee joint. These included terms such as “exoskeleton,” “robotic exoskeleton,” “wearable robot,” “powered orthosis,” and “robotic orthosis,” combined with “knee” or “knee joint,” and terms related to human or clinical testing (eg, “human,” “patient,” “clinical,” “trial,” and “experiment”)*.* Studies that focus solely on simulation, musculoskeletal modeling, finite element analysis, or in silico approaches were excluded at the search level where possible.

Database-specific Boolean search strings were developed and adapted for each platform. The full reproducible search strategies for all databases are provided in Multimedia Appendix 1.

The search was limited to English-language publications due to feasibility constraints in screening and data extraction. Gray literature, trial registries, and industry- or manufacturer-reported studies were excluded because this review focused on peer-reviewed scientific evidence. These decisions may introduce language and publication bias, and this is acknowledged in the limitations.

### Eligibility Criteria

#### Inclusion Criteria

Studies were included if they met the following criteria:

- Investigated knee-focused exoskeletons or robotic knee orthoses designed for gait assistance, rehabilitation, or functional lower-limb activities.
- Involved human participants, including healthy individuals, pediatric populations, or clinical populations with neurological or orthopedic impairments.
- Reported original experimental, observational, feasibility, or clinical trial data and were published as peer-reviewed full-text articles.
- Were published between 2015 and 2025.
- Were available in English.

#### Exclusion Criteria

Studies were excluded if they:

- Articles, systematic reviews, meta-analyses, editorials, protocols, conference abstracts, or opinion papers were reviewed.
- Investigated multijoint exoskeleton (hip-knee, knee-ankle, or full lower-limb exoskeletons) where knee-specific outcomes could not be isolated.
- Focused solely on simulation, finite element modeling, musculoskeletal modeling, or in silico analysis without human validation.
- Investigated devices not related to knee function or gait rehabilitation (eg, upper-limb exoskeletons).
- Lacked sufficient methodological or outcome data for extraction and evaluation.

### Study Process

Following the initial database search, 71 duplicate records were removed, resulting in 2418 unique articles. Study selection was performed in multiple stages.

First, an AI-assisted abstract screening was applied to articles retrieved from Web of Science, IEEE Xplore, PubMed, and Scopus. Screening was performed using the GPT-4 large language model accessed through the OpenAI API. The model was not additionally trained on domain-specific datasets and relied on its pretrained language understanding capabilities. The filtering prompt and classification code were developed by reviewer AZ and applied prior to manual screening (provided in Multimedia Appendix 2). The AI-assisted screening used a prompt-based binary classification approach in which each abstract was evaluated to determine whether the study focused exclusively on knee exoskeletons. The model was instructed to retain studies by default and to exclude articles only when the abstract explicitly involved joints other than the knee (eg, hip, ankle, foot, or multijoint lower-limb systems). In ambiguous cases, studies were retained for further manual evaluation. This conservative filtering strategy was intentionally designed to prioritize sensitivity and minimize false exclusions.

Of the 2418 unique records, 1089 were excluded solely through AI-assisted abstract screening. To validate the performance of the AI-assisted filtering process, a random subset of AI-excluded records was manually reviewed by the authors. This validation confirmed that the majority of excluded studies involved multijoint or non–knee-focused exoskeleton systems. After AI-assisted screening, 71 articles from Web of Science, 21 from IEEE Xplore, 29 from PubMed, and 997 from Scopus remained eligible for subsequent manual screening.

Second, articles retained after AI-assisted screening underwent manual review to confirm eligibility and ensure that the studies focused exclusively on knee exoskeletons and represented original research rather than review articles. Manual screening was performed independently by 4 reviewers (DK, Zeeshan, AN, and ARK). Following AI-assisted and manual screening, 1166 articles were retained for full-text assessment. Full-text screening further evaluated eligibility based on the predefined inclusion and exclusion criteria, resulting in 112 studies selected for data extraction.

### Data Extraction and Quality Assessment

Data extraction was performed for all eligible studies following full-text screening. For each study, the following information was systematically extracted and organized into structured tables: author name and DOI, article title, database source, device name, weight, mechanism type, control system, actuators, range of motion, clinical testing details, and patient type. These data formed the basis for qualitative and comparative analysis of knee exoskeleton technologies and applications.

### Risk of Bias Assessment

The risk of bias was assessed based on the design and methodological characteristics of the included studies. This approach was adopted because the reviewed literature included randomized clinical trials, nonrandomized clinical studies, experimental feasibility studies involving healthy participants, and technical validation studies. Risk of bias was assessed according to the study design: randomized controlled trials were evaluated using the RoB-2 tool. Nonrandomized clinical studies involving patient populations were assessed using the ROBINS-I (Risk of Bias in Nonrandomized Studies of Interventions) tool, which evaluates bias due to confounding, participant selection, intervention classification, deviations from intended interventions, missing data, outcome measurement, and selective reporting. Experimental and feasibility studies conducted in healthy participants were assessed using a modified Downs and Black checklist, as these studies typically focus on technical feasibility, biomechanical performance, usability, or short-term functional outcomes rather than clinical efficacy. Risk-of-bias assessment was independently performed by 3 reviewers (DK, PKJ, and Zeeshan), and disagreements were resolved through discussion. Nonhuman validation studies were not assessed using clinical risk-of-bias tools, as they do not evaluate health outcomes, and were analyzed descriptively with emphasis on device architecture, actuation strategy, sensing configuration, control method, and technical validation outcomes.

Risk-of-bias assessments were incorporated into the narrative synthesis, with greater emphasis placed on findings from studies demonstrating lower risk of bias or higher methodological quality.

## Results

### Study Selection

A total of 32 articles met all inclusion criteria and were included in the final review.

**Figure 1.**
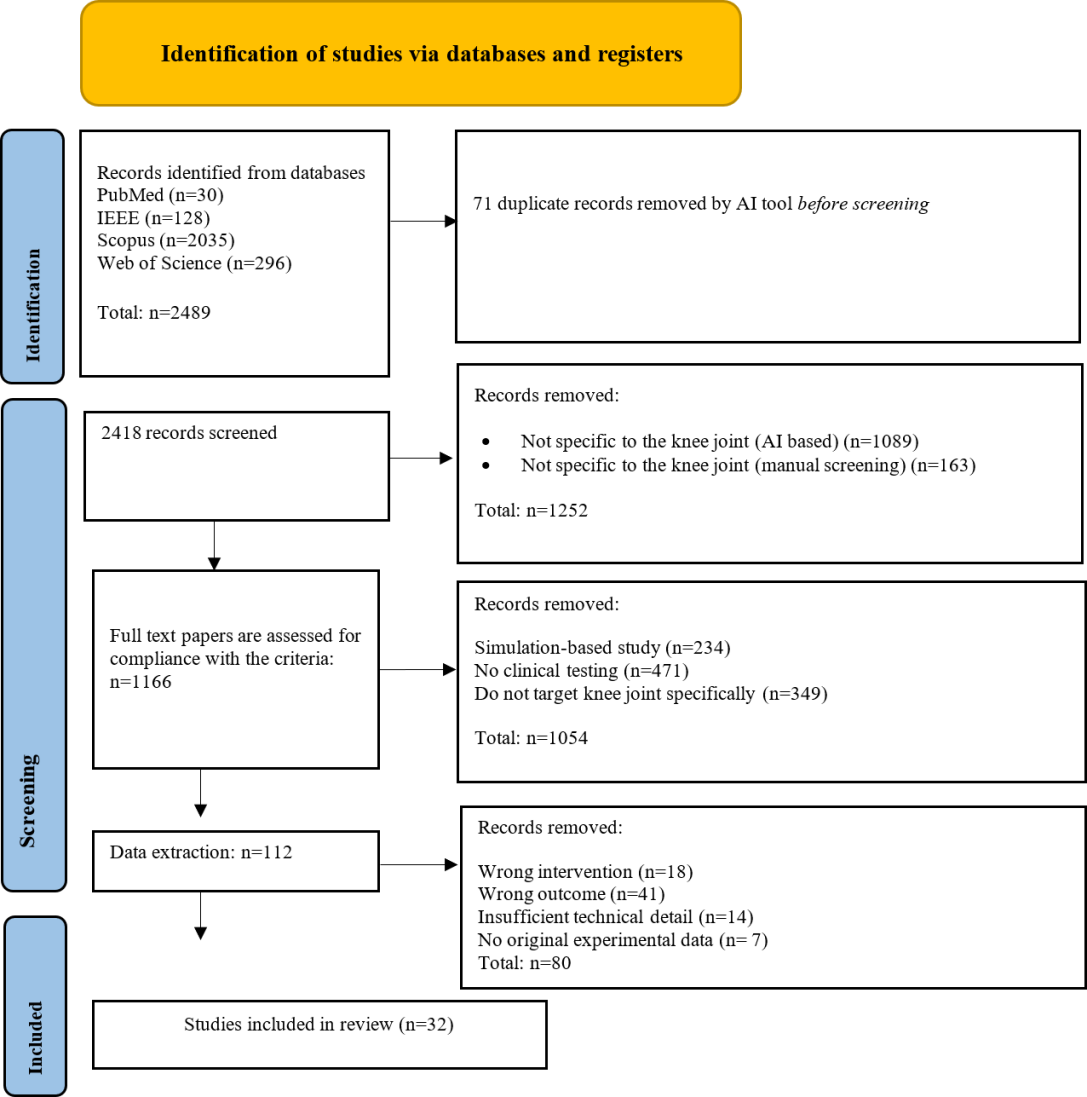
PRISMA (Preferred Reporting Items for Systematic Reviews and Meta-Analyses) flowchart on the selection of papers*.*

Figure 1 illustrates the overall study selection process, detailing the reasons for excluding certain articles and their corresponding numbers.

To reflect the multidisciplinary nature of this field and to ensure clarity in synthesizing the evidence, the Results section was divided into two complementary parts. The first part focuses on the engineering perspective, summarizing design concepts, actuation strategies, and control approaches that enable the functionality of knee exoskeletons (Table 1). The second part addresses the clinical perspective, highlighting patient populations, rehabilitation goals, and functional outcomes associated with the use of knee exostheses (Table 1). This structured approach highlights the importance of integrating engineering advances with clinically relevant outcomes to ensure effective rehabilitation.

**Table 1.** Clinical and engineering characteristics of knee exoskeleton systems.

Author and year	Study design	Device name	Population		Sensor modality	Control architecture	Actuation type	Maximum assistive torque (Nm)
			Group	Sample size				
Pugliese et al, 2025 [27]	Experimental study	Wearable POF^a^ knee-sleeve kinematics estimator	Healthy individuals	31	Polymer optical fiber	Predictive learning-based control	Sensor only	—^b^
Barquín-Santos et al, 2024 [28]	Nonrandomized clinical trial	MAK^c^ robotic device	Postsurgical	6	Joint encoder and torque sensing	Trajectory tracking control	Motor-driven with gearbox	10 to 30
Puyuelo-Quintana et al, 2020 [29]	Cross-sectional study	Portable lower-limb exoskeleton (prototype)	Stroke and Multiple Sclerosis	5	Joint encoders and foot pressure sensors	Gait-phase-based control	Motor-driven with transmission	10 to 25
Monteiro et al, 2024 [30]	Experimental study	Knee exoskeleton with HIL^d^-optimized assistance (name not specified)	Healthy individuals	5	Joint encoder and gait event sensing	Assist as needed adaptive control	Motor-driven	5 to 20
Dai et al, 2024 [31]	Experimental study	Rigid-soft hybrid knee exoskeleton (name not specified)	Healthy individuals	5	Foot pressure sensors, encoders, and inertial sensors	Gait-phase-based control	Motor-driven cable transmission	10 to 30
Zhou et al, 2023 [32]	Experimental study	Passive knee-assisted exoskeleton (spring mechanism)	Healthy individual	1	—	Passive control	Passive	—
Kittisares et al, 2023 [33]	Single-case feasibility study	Dual 4-bar linkage knee exoskeleton	Healthy individual	1	Hydraulic pressure sensing and joint angle sensing	Gait-phase-based control	Hydraulic artificial muscle	15 to 40
Wang et al, 2021 [34]	Experimental study	Adaptive knee exoskeleton with buffering function	Healthy individual	1	Joint angle sensor and motor encoder	Trajectory tracking control	Motor-driven	10 to 30
Kim et al, 2015 [35]	Experimental study	Knee exoskeleton prototype	Healthy individual	1	Foot pressure sensors and knee torque sensors	Gait-phase-based control	Motor-driven with gearbox	5 to 20
Villa-Parra et al, 2018 [36]	Experimental study	sEMG^e^-controlled knee exoskeleton	Healthy individuals	12	Surface electromyography and joint encoder	Bio-signal proportional control	Motor-driven	5 to 20
Chen et al, 2018 [37]	Benchtop validation study	Wearable pediatric robotic knee exoskeleton	No human participants	—	Joint encoders, foot pressure sensors, and inertial sensors	Gait-phase-based control	Motor-driven with transmission	5 to 15
Wang et al, 2018 [38]	Benchtop validation study	Lightweight backdrivable knee exoskeleton	No human participants	—	Joint encoder and torque sensing	Force and impedance control	Motor-driven low-ratio transmission	5 to 20
Jammeli et al, 2021 [39]	Experimental study	Knee rehab exoskeleton with explicit MPC^f^	Healthy individuals	3	Joint encoder and velocity estimation	Predictive learning-based control	Motor-driven	10 to 30
Park et al, 2020 [40]	Experimental study	Hinge-free tethered knee exosuit	Healthy individuals	6	Cable tension sensing and gait-phase detection	Gait-phase-based control	Motor-driven tendon system	5 to 15
Mosconi et al, 2024 [41]	Experimental study	Knee exoskeleton interaction testbed	Healthy individual	1	Joint encoder and torque sensor	Force and torque control	Motor-driven	5 to 30
Rodríguez-Fernández et al, 2022 [42]	Randomized, crossover clinical trial	Knee-powered exoskeleton and knee-ankle-foot orthoses	Spinal cord injury	10	Joint encoders and gait-phase sensing	Gait-phase-based control	Motor-driven	10 to 40
Kittisares et al, 2020 [43]	Experimental study	Four-bar linkage + HAM^g^ knee support device	Healthy individual	1	Hydraulic pressure sensing and angle measurement	Low-level control	Hydraulic artificial muscle	15 to 40
Lora-Millan et al, 2023 [44]	Nonrandomized clinical trial	REFLEX knee exoskeleton	Poststroke hemiparetic	7	Joint encoders, inertial sensors, and foot pressure sensors	Assist-as-needed adaptive control	Motor-driven	10 to 30
Velasco-Guillen et al, 2024 [45]	Experimental study	Torque-assisted knee exoskeleton with elastic fault compensation	Healthy individual	1	Joint encoder and elastic torque sensing	Compliance and fault management	Motor-driven series elastic actuator	10 to 30
Zhang et al, 2024 [46]	Experimental study	Unilateral active knee exoskeleton with intention recognition	Stroke patients with hemiplegic paralysis	3	Inertial sensors, foot pressure sensors, and joint encoders	Predictive learning-based control	Motor-driven	5 to 25
Olinski, 2025 [47]	Experimental study	Knee joint exoskeleton prototypes	Healthy individuals	2	Kinematic measurement systems	Low-level control	Motor-driven or passive	10 to 30
Zhang et al, 2023 [48]	Experimental study	Neuromusculoskeletal model-informed machine learning–based control of a knee exoskeleton	Healthy individuals	8	Kinematic sensing	Predictive learning-based control	Motor-driven	5 to 25
Hidayah et al, 2021 [49]	Experimental study	SpringExo is a coil-spring design that aids in knee extension	Healthy individuals	7	External motion capture and force measurement	Passive control	Passive elastic element	—
Eveld et al, 2023 [21]	Experimental study	Stumble-recovery knee exoskeleton	Healthy individuals	3	Inertial sensors, joint encoder, and foot sensors	Gait-phase-based control	Motor-driven	15 to 40
Shideler et al, 2020 [50]	Experimental study	Pediatric hybrid exoskeleton + noninvasive Neuromuscular Electrical Stimulation	A teenager with bilateral spastic CP^h^	1	Gait-phase sensing and stimulation control	Bio-signal proportional control	Electrical stimulation and motor-driven	5 to 15
Lerner et al, 2017 [51]	Nonrandomized clinical trial	Lower-extremity exoskeleton for crouch gait	Children with CP	7	Joint encoders and gait event sensing	Gait-phase-based control	Motor-driven	5 to 15
Ben-David et al, 2022 [52]	Experimental study	Passive knee exoskeleton for jumping	Healthy individuals	10	—	Passive control	Passive	—
Goffredo et al, 2022 [53]	Observational study	Exoskeleton used for community ambulation (name not specified)	Healthy individuals	5	Device encoders	Gait-phase-based control	Motor-driven	10 to 40
Sarkisian et al, 2021 [54]	Experimental study	Powered knee exoskeleton with self-aligning mechanism	Healthy individuals	14	Joint encoder and interaction force sensing	Force and impedance control	Motor-driven	10 to 30
Wang et al, 2023 [55]	Experimental study	Knee flexion-assisted human-exoskeleton system	Healthy individuals	7	Joint encoder and gait-phase sensing	Gait-phase-based control	Motor-driven	5 to 20
Wang et al, 2023 [56]	Experimental study	Rigid-soft hybrid knee exoskeleton	Healthy individuals	6	Joint encoder inertial sensors and foot pressure sensors	Gait-phase-based control	Motor-driven cable transmission	10 to 30
de Miguel Fernández et al, 2023 [57]	Nonrandomized clinical trial	Knee exoskeleton for adapted assistance/resistance training	Poststroke individuals	6	Joint encoder inertial sensors and foot pressure sensors	Force and impedance control	Motor-driven	10 to 30

a POF: polymer optical fiber.

b Not applicable.

c MAK: Marsi active knee.

d HIL: human-in-the-loop.

e sEMG: surface electromyography.

f MPC: model predictive control.

g HAM: hydraulic artificial muscle.

h CP: cerebral palsy.

### Part 1: Engineering Perspective

#### Actuation Systems

The choice of actuator in knee exoskeletons is constrained by the trade-off between high-fidelity torque control and the penalties of increased mass, complexity, and reduced portability. Three prevailing actuation paradigms can be identified within this design space: motor-driven rigid joints, compliant or distributed actuation systems, and passive elastic mechanisms.

**Figure 2.**
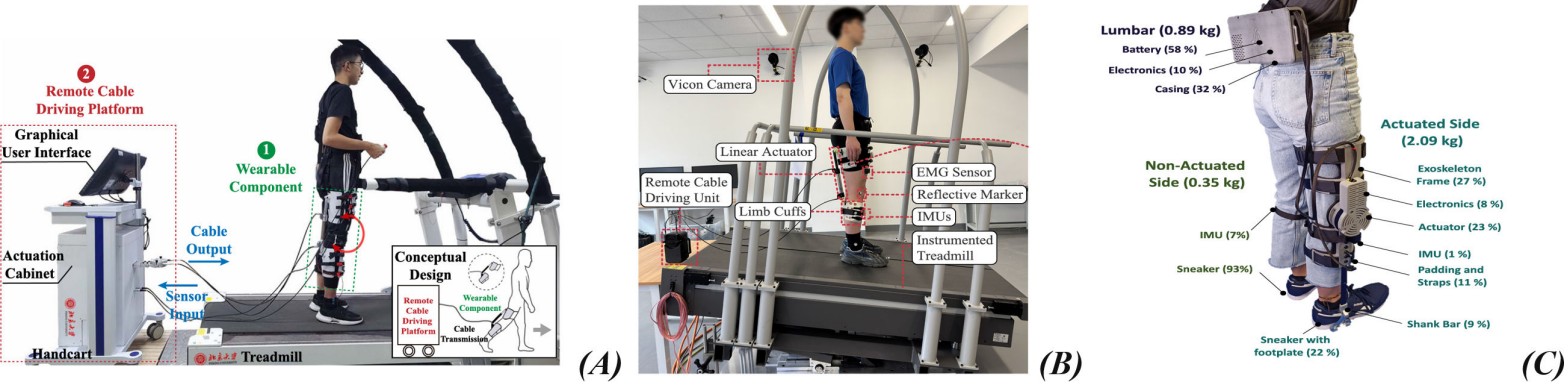
Representatives of different actuation architectures in knee exoskeletons: (A) a rigid motor-driven powered exoskeleton using electric motor actuation with gearbox transmission for precise torque control [56], (B) a rigid-soft hybrid exoskeleton combining a rigid frame with compliant elements to improve comfort and reduce joint misalignment [31], and (C) a passive knee exoskeleton using coil-spring mechanisms to store and return elastic energy during gait without external power [52]*.* EMG: electromyography; IMU: inertial measurement unit.

The most common solution has been electric motor-driven actuation for rehabilitation-focused devices and gait-assist, one of which is shown in Figure 2A. In such systems, direct current or brushless direct current motors are typically used, with gear reductions used to provide repeatable, controllable knee torques, which can be used in closed-loop torque or impedance control structures [29,34,38,42,56]. It provides accurate command tracking and can be easily combined with sensor-based feedback required for safe interactions during rehabilitation activities. In the reviewed literature, assistive torques typically range from 5 to 30 Nm, which is consistent with the mechanical loads of level walking and early rehabilitation [29,31,36,44,56]. Stair-climbing or perturbation-recovery devices are designed for more challenging tasks that require much higher torque capability, with peak assistive torques of nearly 40 Nm [21,33]. They usually accept larger actuators, higher transmission gears, and external power. To dampen interaction forces in high-impedance motorized joints, several papers have added compliance to the actuation chain, either in series-elastic or mechanically compliant form. This was done to enhance shock resistance and the accuracy of torque regulation [45,54].

By contrast, a second design focuses on interaction quality and wearability with compliant or distributed actuation. Examples of such solutions include hybrid rigid-soft exoskeletons and hinge-free textile-based exosuits, which redistribute mass closer to the interface, as shown in Figure 2B [31]. The objects of these systems maintain natural knee kinematics and reduce discomfort associated with joint misalignment. Although compliant actuation is less disruptive and less damaging, it presents engineering challenges, including transmission and friction losses and a more complex calibration process.

The third paradigm comprises passive knee exoskeletons that store and recover elastic energy without external power or closed-loop control. Assistance during certain stages of motion is provided by coil springs and compliant mechanisms, as shown in Figure 2C, thereby reducing device mass and simplifying the mechanism [32,49,52]. These systems have the benefit of being portable and low-complexity, but do not offer flexibility across users and tasks.

The smaller group of papers investigated hydraulic artificial muscles as an alternate actuation concept, also driven by their high force density and natural compliance, especially when a high torque is needed in large knee flexion angles [33,43]. Given these merits, fluidic actuation systems introduce considerable complexity in pressure and power control. They can only be applied to tethered concept prototypes, thereby hampering translational scalability.

#### Sensor Design and Engineering Integration

**Figure 3.**
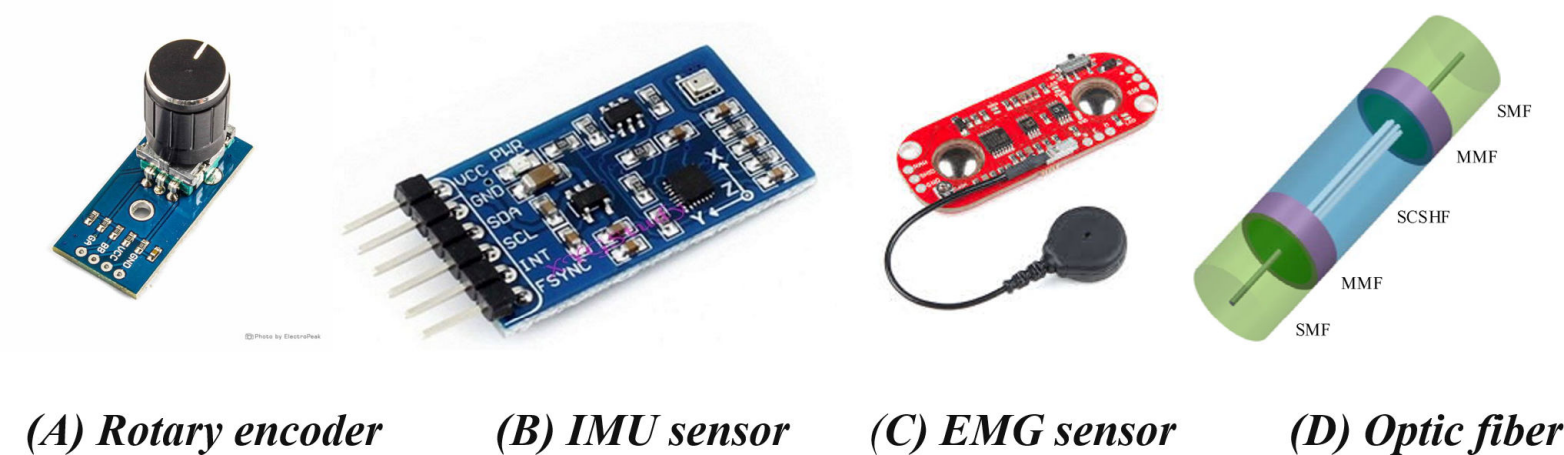
Examples of sensor modalities integrated into knee rehabilitation exoskeletons: (A) rotary encoder mounted at the joint axis for high-resolution knee angle measurement [58], (B) inertial measurement unit (IMU) attached to the leg segment for gait-phase detection and swing-stance classification [59], (C) surface electromyography (sEMG) electrodes placed over the quadriceps for real-time motor intention estimation [60], and (D) polymer optical fiber (POF) embedded in a textile sleeve for nonrigid kinematic sensing [61]. MMF: multimode fiber; SCSHF: single-core six-hole microstructure optical fiber; SMF: single-mode fiber.

All powered knee exoskeletons have joint-level kinematic sensing as the basic sensing layer. Rotary encoders, as shown in Figure 3A, in the actuation or transmission system can provide high-resolution knee angle measurements needed to control closed-loop torque, knee position, or impedance [34,38,39,54]. In engineering terms, encoder-based sensing operates in a high-bandwidth, low-latency range, but its accuracy also depends on mechanical alignment. Various studies underscore that encoder measurements reflect device joint motion rather than the actual motion of the anatomy, making them susceptible to joint-axis misalignment and soft-tissue deformation.

Most systems use gait-phase detectors based on inertial measurement units (IMUs), shown in Figure 3B, and plantar pressure sensors to provide task-oriented support, particularly while walking. IMUs provide the segment’s angular velocity and orientation, thereby supporting the detection of swing-stance transitions across different walking speeds [29,31,46,56]. Plantar pressure sensors or foot switches provide temporally accurate event information, for example, heel strike and toe-off, and are often used with IMU data to enhance robustness and minimize false state changes [35,46]. The current success of multimodal gait sensing has been a companion to recognition throughout the field due to the limited reliability of single-sensor modalities across conditions, particularly for impaired gait patterns.

A smaller body of related work uses surface electromyography (EMG) to infer purpose and control assistance in real time [36,48,50]. In engineering terms, EMG, shown in Figure 3C, provides an entirely new control signal that occurs before joint motion and, in theory, may enable predictive assistance. There are some practical difficulties identified with the implementation of EMG, such as electrode placement sensitivity, signal variability due to fatigue or perspiration, and susceptibility to motion artifacts. These properties require complex signal-processing pipelines and frequent recalibration, thereby restricting the use of EMG-based sensing to a laboratory setting.

The third way is an emerging technique in nonrigid, textile-based sensing technologies to overcome the comfort and alignment constraints of solid sensor attachment. The use of polymer optical fiber-based strain sensors, shown in Figure 3D, in wearable sleeves indicates that it is possible to measure knee kinematics without placing mechanical limits on the joint [27]. Although these methods make wearable objects less sensitive to alignment and motion tracking, they are currently used predominantly for motion detection rather than for closed-loop actuation.

#### Control Strategies and Algorithms

The control strategies for knee exoskeletons have been designed to balance accuracy in assistance timing, stability in interactions, and computational viability against real-time requirements. In the literature reviewed, control architectures are always hierarchical, with high-level task or gait-state estimation.

A finite-state machine (FSM)–controlled walking aid is most often implemented for gait-phase-driven control. FSMs allow deterministic, phase-specific assistance modulation in stance and swing, and conform control output to the gait cyclic pattern, with minimal computational cost [29,31,42,46,48]. The method is quite appealing for rehabilitation because the duration of assistance can be predicted. However, FSM-based control is very sensitive to both the correctness and precision of state transitions; misclassifying the gait phase will cause assistance at the wrong time and result in poor-quality interaction.

Some studies use assist-as-needed control strategies, in which the level of assistance is regulated based on the user’s interaction forces or task execution [37,39,57]. From an engineering perspective, these solutions are based on the localization of control complexity. That is, a way of state timing, which necessitates stable low-level controllers and robust sensing. Although assist-as-needed paradigms provide enhanced customization, they are mostly done in a heuristic manner, and few formal reports on stability indicators have been described in the studies reviewed.

**Figure 4.**
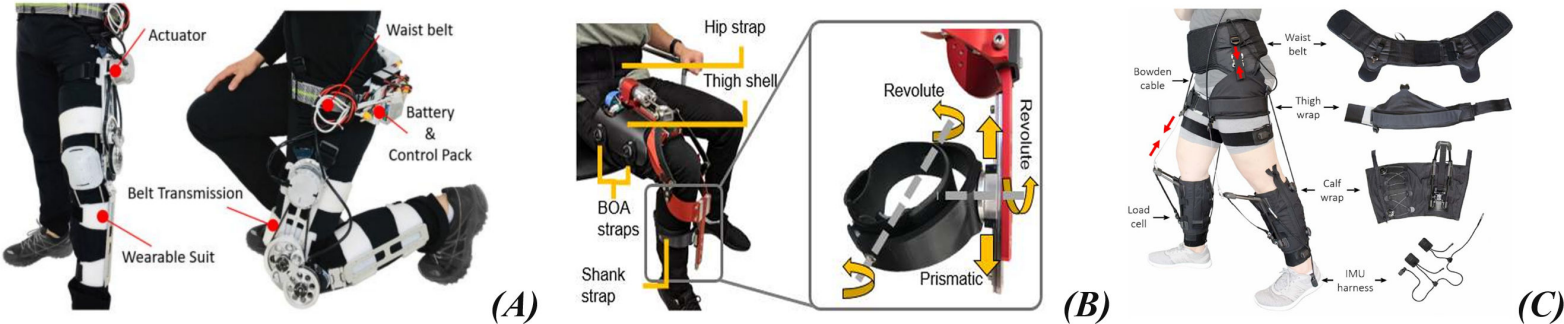
Representatives of different structural design and alignment strategies in knee exoskeletons: (A) a lightweight, back drivable knee exoskeleton featuring a low-ratio transmission for improved transparency and reduced distal inertia [38], (B) a powered knee exoskeleton with a self-aligning mechanism designed to accommodate anatomical variation and reduce parasitic joint forces [54], and (C) a hinge-free tethered exosuit that replaces rigid linkages with textile-based cable routing, preserving natural knee kinematics [40]. BOA: BOA system straps; IMU: inertial measurement unit.

Knee exoskeletons are dominated by low-level torque- and impedance-based control. Torque monitoring enables direct regulation of assistive moments and is applicable to systems with built-in torque sensors or series compliance [34,45]. Instead, we use impedance control, which is commonly used to reduce interaction forces caused by joint misalignment and to maintain back drivability, particularly in comfort-based designs as depicted in Figure 4A,B [38,54]. As illustrated in Figure 4C, the hinge-free exosuit redistributes actuation forces through Bowden cable routing anchored to textile thigh and calf wraps, with a load cell and IMU harness enabling force and kinematic sensing without imposing any rigid constraint on the knee joint axis. Though it has less control bandwidth, it offers the benefit of perceived transparency, at the cost of achievable responsiveness in high-torque or high-speed processes.

More complex control techniques, such as explicit model predictive control, human-in-the-loop optimization, and machine-learning-based controllers, are reported in fewer studies [30,39,48]. The same strategies are expected to enhance adaptability by incorporating predictive models and iterative optimization. They have shown promising results in controlled experiments but are only practical when model-dependent, computationally expensive, and require large amounts of calibration or training data.

#### Engineering Limitations and Challenges

Although substantial advances have been made in developing knee exoskeletons, the reviewed articles indicate a range of inherent engineering issues that limit their effectiveness, wearability, and translational appropriateness. Such restrictions are not limited to designs but are repeated as systems of the subsystems of actuation, sensing, control, and human-robot interface.

One of the major limitations is the mechanical misalignment between the exoskeleton joint and the anatomical knee axis, which increases discomfort and reduces the efficacy of assistance. In systems with tuned joint-level sensory and torque control, the error is due to soft-tissue deformity and anatomical differences in residual limbs. Multiple attempts to reduce these effects include self-aligning or compliant interfaces, but these solutions introduce additional mechanical complexity and mass and represent an open-ended trade-off between alignment strength and structural simplicity [54].

Another prevailing challenge is the mass and inertia of devices. Exoskeletons with motorized knees usually require greater mass and battery capacity, leading to higher distal inertia and reduced user acceptance. To deliver mass in proximity and enhance wearability, hybrid rigorous-soft architecture and remote actuation strategies are currently being explored, but these methods have their own problems: transmission friction, hysteresis, and sensitivity to environmental conditions make repeatable torque delivery difficult to achieve [31,40,56].

**Figure 5.**
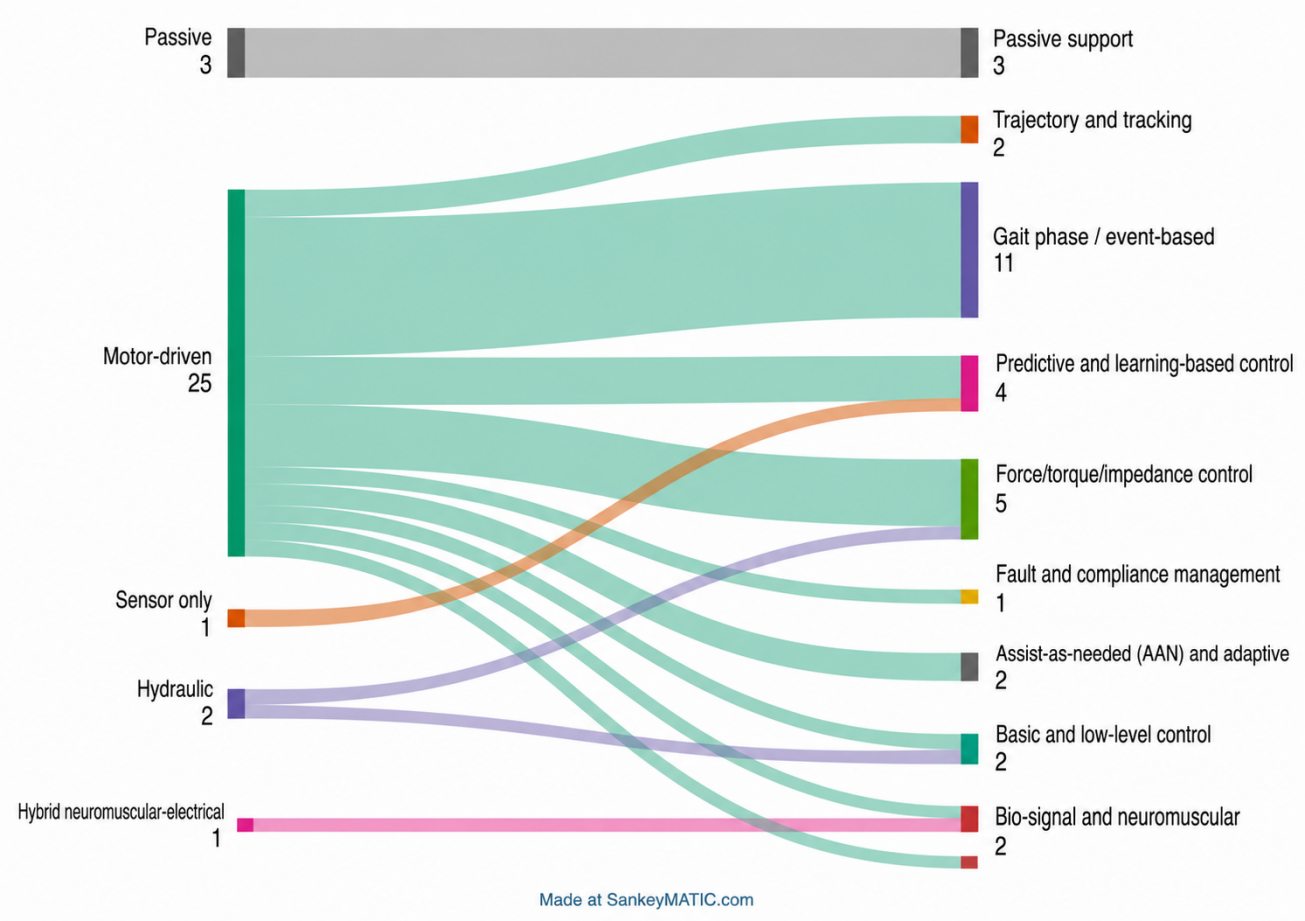
Sankey diagram illustrating the relationship between actuation mechanisms and control architectures across the included knee exoskeleton studies. Arrow width is proportional to the number of studies using each combination. Motor-driven actuation is linked to the widest range of control strategies, reflecting its prevalence and versatility in the reviewed literature. Diagram created using SankeyMATIC [62]*.*

Among other observations, one major finding is that the choice of actuation implicitly limits the entire system architecture. Figure 5 demonstrates that motor-driven systems prevail in the field, and the most diverse control strategies are supported. This is an indication of the predictability and controllability of the electric motors, as well as an indicator that designers are willing to trade the added mass, inertia, and alignment sensitivity to gain the ability to predict the torque generation. Other actuation ideas, such as compliant, passive, or hydraulic actuation, are much less common because they introduce new issues related to controllability, scalability, or power control [34,42]. This implies that the design of knee exoskeletons is constrained by the challenge of achieving both high torque authority and wearability within a single system.

Limitations about sensing also limit the reliability of control. Encoder-based joint sensing quantifies device motion rather than anatomy motion and is thus sensitive to alignment errors, whereas IMU-based gait phase estimation is prone to errors in abnormal gait patterns. All these concerns are part of the problem of the absence of standard benchmarks for sensing accuracy, latency, and durability under realistic operating conditions.

These sensing and mechanical constraints are closely linked to control challenges. FSM-based gait controllers offer robustness and simplicity but lack flexibility when gait patterns deviate from nominal cycles. On the other hand, adaptive and learning-based controllers impose additional computational and modeling constraints that are likely to compromise real-time reliability and safety. It is worth noting that there is limited research on formal stability assessment or long-term assessment, and there remains an open question regarding the performance of controllers when fatigued, affected by sensor drift, or used over long periods of the day [30,39,48].

Lastly, the absence of engineering standards impedes meaningful comparisons across studies. Measures such as energy efficiency, long-term functionality, and durability are not commonly addressed, even though they directly affect practical use. In the absence of regular evaluation of these factors, it remains difficult to estimate the effectiveness of these design trade-offs in nonexperimental settings.

To conclude, engineering issues include energy efficiency and long-term operability. Battery capacity, power consumption, and thermal management are rarely measured in standardized units, even though they directly influence portability and user compliance.

#### Risk of Bias Quality

**Figure 6.**
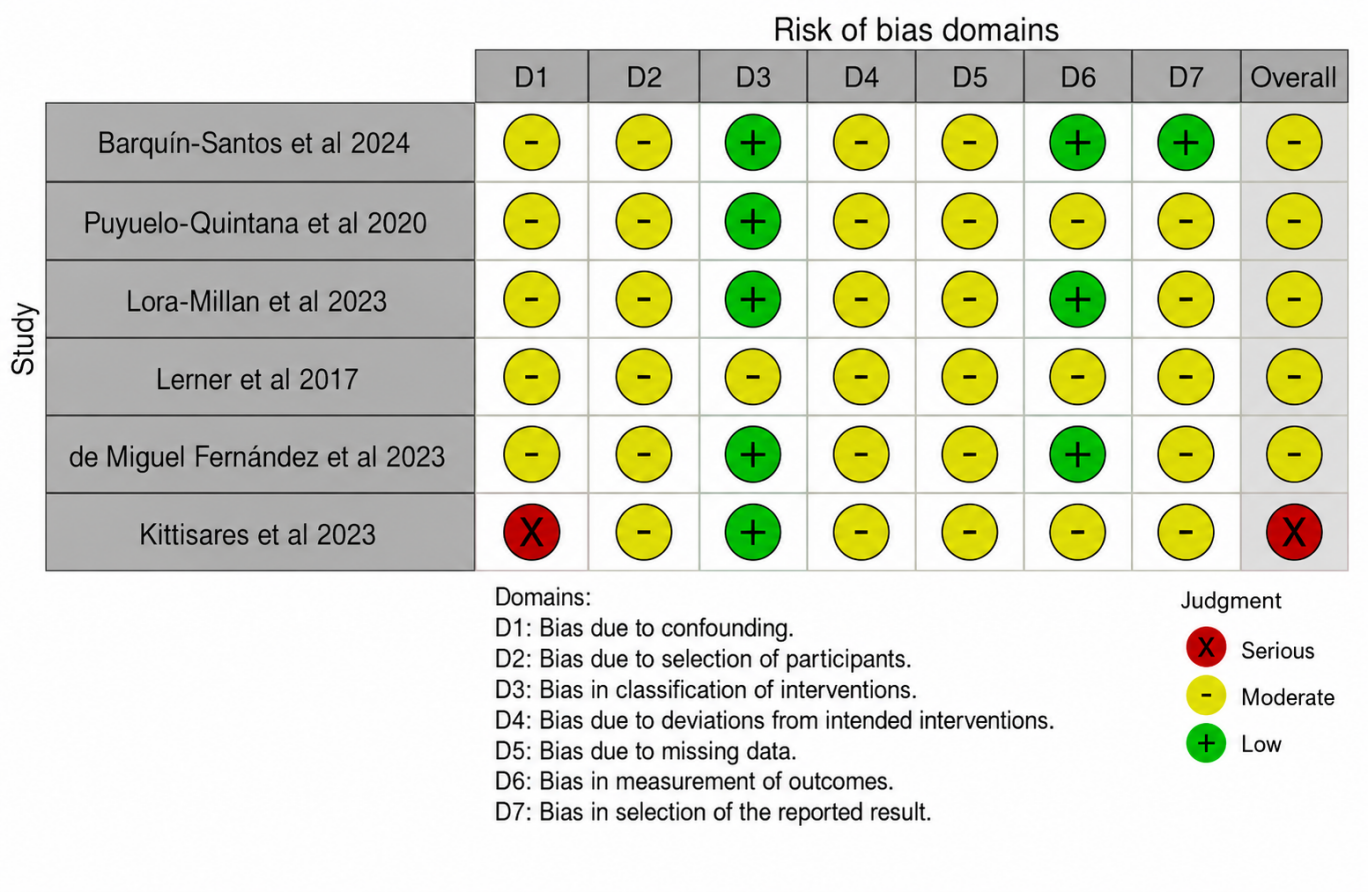
ROBINS-I traffic light plot for nonrandomized clinical studies. Risk of bias assessment of nonrandomized clinical studies using the ROBINS-I tool [28,29,33,44,51,57]. Created using the Robvis app. ROBINS-I: Risk of Bias in Nonrandomized Studies of Interventions.

**Figure 7.**
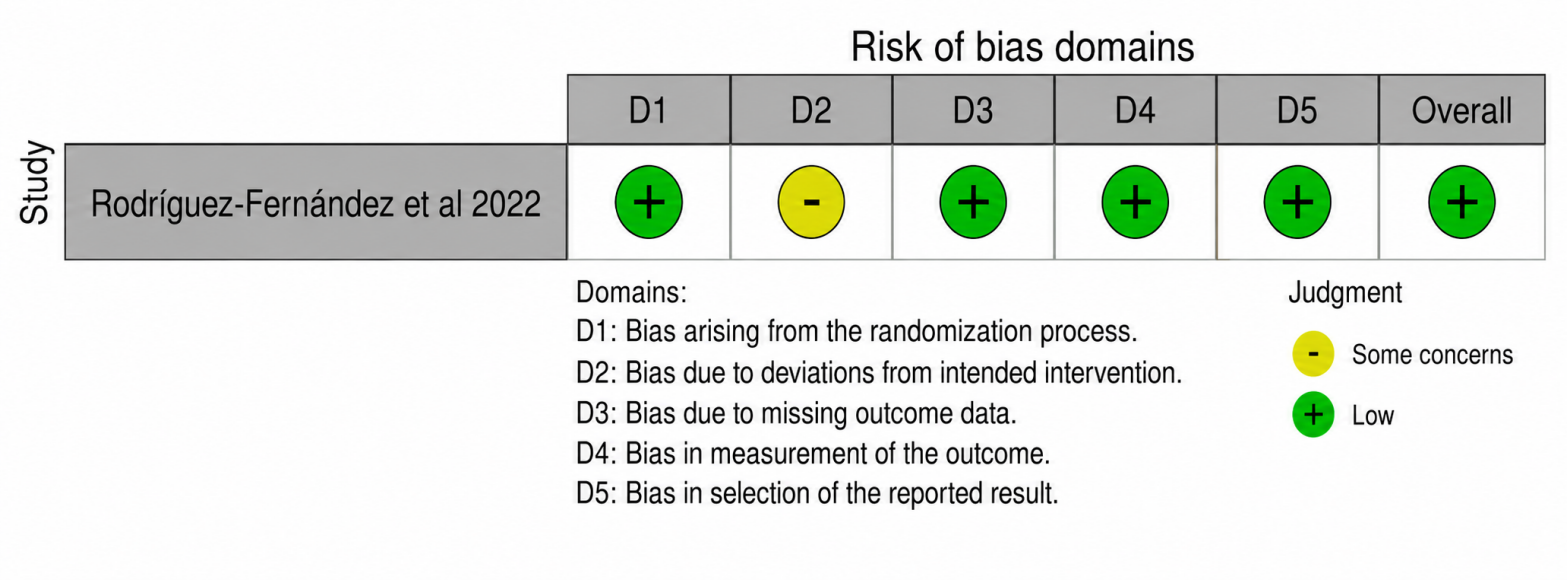
RoB-2 traffic light plot for randomized controlled trials. Risk of bias assessment of the randomized crossover clinical trial using the Cochrane Risk of Bias 2 (RoB-2) tool [42]. Created using the Robvis app.

Risk of bias and methodological quality were assessed according to study design using the Cochrane RoB-2 tool [63] for randomized controlled trials, ROBINS-I [64] for nonrandomized clinical studies, and a modified Downs and Black checklist [65] for experimental and feasibility studies (Figures 6 and 7, Table 2). Among the nonrandomized clinical studies assessed using ROBINS-I, most were rated as having a moderate risk of bias [28,29,44,51,57] (Figure 6). The most common sources of bias were confounding, participant selection, and lack of blinding during intervention delivery and outcome assessment. One study was classified as having a serious risk of bias due to substantial methodological limitations and potential confounding [33]. The randomized crossover clinical trial by Rodríguez-Fernández et al [42] was rated as having an overall low risk of bias according to the RoB-2 tool (Figure 7). However, some concerns were identified regarding deviations from intended interventions, as participant blinding was not feasible in wearable exoskeleton studies.

**Table 2.** Methodological quality assessment of experimental and feasibility studies using the modified Downs and Black checklist. Scoring was conducted using the 27-item modified Downs and Black checklist, encompassing the domains of reporting quality, external validity, internal validity (bias and confounding), and statistical power [65].

Study	Reporting	External validity	Bias	Confounding	Power	Total	Quality
Pugliese et al, 2025 [27]	8	1	4	3	0	16	Fair
Monteiro et al, 2024 [30]	9	1	5	4	0	19	Good
Dai et al, 2024 [31]	9	1	5	4	0	19	Good
Villa-Parra et al, 2018 [36]	8	1	4	3	0	16	Fair
Jammeli et al, 2021 [39]	8	1	4	3	0	16	Fair
Park et al, 2020 [40]	8	1	4	3	0	16	Fair
Mosconi et al, 2024 [41]	7	1	4	3	0	15	Fair
Velasco-Guillen et al, 2024 [45]	8	1	4	3	0	16	Fair
Olinski et al, 2025 [47]	7	1	4	3	0	15	Fair
Zhang et al, 2023 [48]	8	1	4	3	0	16	Fair
Hidayah et al, 2021 [49]	8	1	4	3	0	16	Fair
Eveld et al, 2023 [21]	8	1	5	3	0	17	Fair
Ben-David et al, 2022 [52]	9	1	5	4	0	19	Good
Sarkisian et al, 2021 [54]	8	1	4	3	0	16	Fair
Wang et al, 2023 [55]	8	1	4	3	0	16	Fair
Wang et al, 2023 [56]	8	1	4	3	0	16	Fair
Kittisares et al, 2023 [33]	8	1	4	3	0	16	Fair
Wang et al, 2021 [34]	8	1	4	3	0	16	Fair
Kim et al, 2015 [35]	8	1	4	3	0	16	Fair
Zhou et al, 2023 [32]	8	1	4	3	0	16	Fair
Kittisares et al, 2020 [43]	8	1	4	3	0	16	Fair
Zhang et al, 2024 [46]	8	1	4	3	0	16	Fair
Goffredo et al, 2022 [53]	7	1	4	3	0	15	Fair
Shideler et al, 2020 [50]	8	1	4	3	0	16	Fair

Experimental and feasibility studies evaluated using the modified Downs and Black checklist generally demonstrated fair-to-good methodological quality. Total scores ranged from 15 to 19 points (Table 2). Three studies achieved “good” (19 points) quality ratings [30,31,52], whereas the remaining studies were classified as “fair.” Overall, studies consistently provided detailed descriptions of exoskeleton architecture, control strategies, sensor integration, and outcome measures. However, important methodological limitations were identified. External validity was often limited because most studies used small convenience samples, healthy participants, or single-subject feasibility designs, thereby reducing generalizability to broader clinical populations. Randomization and blinding were generally not feasible. In addition, none of the included experimental studies reported formal power calculations or sample-size justification, indicating a persistent risk of bias in the statistical power domain.

Two studies involving benchtop or no human-validation experiments were not assessed using formal clinical risk-of-bias tools because they did not evaluate patient outcomes or clinical interventions [37,38]. Instead, these studies were analyzed descriptively with emphasis on technical reporting quality, actuator validation, sensor integration, and controller performance. Both studies provided detailed engineering descriptions and reproducible experimental protocols; however, their findings remain limited to laboratory validation settings and cannot be generalized to clinical rehabilitation outcomes.

### Part 2: Clinical Perspective

#### Study Populations and Clinical Settings

This section summarizes the clinical, biomechanical, and user-oriented outcomes of knee exoskeletons for gait improvement and rehabilitation. The presented studies span orthopedic, neurological, pediatric, and healthy populations and investigate both powered and passive knee exoskeleton systems across laboratory, clinical, and real-world settings.

Among the included studies, 22 studies were conducted in healthy adult participants, primarily to evaluate feasibility, safety, biomechanical effects, and control strategies. Clinical populations were investigated in 8 studies: neurological populations represented the largest clinical subgroup and included individuals with stroke, multiple sclerosis, spinal cord injury, and hemiplegic paralysis [29,42,44,46,57]. Orthopedic applications were limited to patients in the early postoperative phase after total knee arthroplasty [28], whereas pediatric populations included children and adolescents with cerebral palsy, crouch gait, or knee-extension deficits [50,51]. In addition, two studies were conducted without human participants [37,38].

#### Neurological Rehabilitation Outcomes

Knee exoskeletons were most frequently used in neurological gait rehabilitation. In stroke and multiple sclerosis populations, portable, powered knee exoskeletons have been shown to be feasible and safe, with participants able to complete overground walking tasks without major adverse events [29]. Improvements were reported in spatiotemporal gait parameters and walking performance, although responses varied across individuals and control strategies.

In a randomized crossover clinical trial involving individuals with spinal cord injury, a knee-powered exoskeleton demonstrated improved walking efficiency compared with conventional knee-ankle-foot orthoses, highlighting the potential of active knee assistance to reduce ambulatory effort [42]. Poststroke clinical trials have reported improvements in gait control and adaptability when assistance levels are adjusted to task demands or individual user characteristics, particularly during the stance and swing phases of gait [44,57]. Swing phase–specific assistance was associated with increased knee flexion and improved foot clearance during walking in patients with hemiplegic stroke [17].

#### Orthopedic and Postsurgical Applications

Orthopedic applications were primarily represented by a clinical trial involving patients undergoing early rehabilitation following total knee arthroplasty. In this study, the integration of a robotic knee device within 48 hours postsurgery was reported to be safe and well-tolerated [28]. Participants successfully completed assisted exercises, including passive mobilization, sit-to-stand transitions, and gait training, with no significant adverse events reported. Patient satisfaction was high, and clinical outcomes were comparable to those achieved with conventional rehabilitation protocols, suggesting that knee exoskeletons may serve as a feasible adjunct during early postoperative recovery. However, despite these promising preliminary findings, validated clinical evidence for orthopedic and postsurgical applications of knee exoskeletons remains extremely limited. Most of the currently available studies are feasibility-focused, involve small sample sizes, and lack long-term follow-up or controlled, comparative designs. In addition, there remains insufficient data on other orthopedic conditions, indicating a significant gap in the current literature and a need for larger clinical trials for specific conditions.

#### Pediatric Rehabilitation and Gait Correction

Pediatric studies primarily addressed pathological gait patterns, particularly crouch gait and impaired knee extension in children and adolescents with cerebral palsy. Powered knee exoskeletons were associated with clinically meaningful improvements in knee extension during stance and reductions in crouch gait severity [51]. In a case-based study, combining mechanical knee assistance with noninvasive neuromuscular electrical stimulation led to greater improvements in knee extension than exoskeleton assistance alone, highlighting the potential benefits of multimodal rehabilitation strategies in pediatric populations [50].

#### Functional Tasks and Outcome Domains

Across the included studies, level walking was the most assessed functional task, evaluated in both healthy and clinical populations [29,42]. Additional tasks included stair ascent [33], sit-to-stand transitions [28], squatting [49], stumble recovery [21], jumping [52], and community ambulation in outdoor environments [53].

Clinical outcome measures clustered into four main domains:

1. Gait performance and biomechanics, including walking speed, knee range of motion, joint moments, and stance- and swing-phase characteristics [29,31,51].
2. Neuromuscular outcomes, such as muscle activation amplitude, coordination, and interaction torque, reflecting reductions in muscular demand during assisted walking [36,41,50].
3. Physiological and effort-related outcomes, including metabolic cost and fatigue, with several studies reporting reduced energetic demand during exoskeleton-assisted gait [30,40,49].
4. User-centered outcomes, including comfort, safety, stability, alignment, and perceived trust, are particularly emphasized in studies addressing interface design and migration resistance [45,54].

### Relationship Between Engineering Design and Clinical Outcomes

Clinical and biomechanical outcomes were frequently associated with specific engineering design characteristics across the included studies, as shown in Table 3. Motor-driven exoskeletons with gait-phase-based control were among the most commonly investigated systems in neurological rehabilitation and were associated with improvements in gait coordination, swing-phase assistance, and walking support in individuals with stroke and spinal cord injury [29,42,44,57]. Similarly, assist-as-needed and adaptive control strategies were developed to modulate assistance in response to user performance and task demands, thereby supporting individualized rehabilitation approaches [48,57].

**Table 3.** Relationship between engineering design features and reported clinical or biomechanical outcomes.

Engineering feature or technical configuration	Reported clinical or biomechanical outcome	Representative studies
Gait-phase-based motor control	Improved gait timing, coordination, and assistive synchronization	Kim et al, 2015 [35]; Wang et al, 2023 [55]
Assist-as-needed and adaptive control	Personalized rehabilitation and task-specific gait assistance	Zhang et al, 2023 [48], de Miguel Fernández et al, 2023 [57]
Passive elastic actuation	Reduced muscular effort and assistance during repetitive movement	Hidayah et al, 2021 [49], Ben-David et al, 2022 [52]
High-torque motor-driven systems	Improved support during rehabilitation and pathological gait assistance	Kittisares et al, 2020 [43], Rodríguez-Fernández et al, 2022 [42]
Hybrid rigid-soft structures	Reduced joint misalignment and improved comfort during walking	Wang et al, 2023 [56]
Self-aligning and polycentric knee mechanisms	Improved biomechanical alignment and preservation of physiological knee kinematics	Sarkisian et al, 2021 [54], Olinski et al, 2025 [47]
EMG^a^-driven intention recognition	Improved synchronization between user intention and robotic assistance	Villa-Parra et al, 2018 [36], Zhang et al, 2023 [48]
NMES/FES^b^-triggered robotic assistance	Increased voluntary muscle activation and enhanced motor relearning potential	Shideler et al, 2020 [50]
Assistance/resistance multimodal training	Improved peak knee flexion and gait endurance after stroke rehabilitation	de Miguel Fernández et al, 2023 [57]
IMU^c^-based outdoor gait monitoring	Improved evaluation of community ambulation and ecological walking behavior	Goffredo et al, 2022 [53]
Cable-driven lightweight exoskeleton systems	Improved comfort and reduced restriction of natural gait motion	Wang et al, 2023 [56]
Adaptive oscillator-based gait prediction	Improved real-time gait synchronization and assistive timing	Wang et al, 2023 [55]

a EMG: electromyography.

b NMES/FES: neuromuscular electrical stimulation/functional electrical stimulation.

c IMU: inertial measurement unit.

Passive and compliant exoskeleton systems have been commonly investigated for repetitive movement assistance and the reduction of muscular effort in healthy individuals [40,49,52]. However, these systems generally provided lower torque output and limited adaptability for pathological gait correction. In contrast, higher-torque motorized systems (10‐40 Nm) were more frequently used for stair climbing, perturbation recovery, and poststroke rehabilitation tasks that require greater movement support and stabilization [21,33,43].

Several studies also emphasized the importance of wearable comfort, joint alignment, and interface compliance for user acceptance and stability during assisted gait [45,54]. Hybrid rigid-soft architectures, self-aligning mechanisms, and hinge-free exosuit designs were specifically developed to reduce joint misalignment and preserve more physiological gait kinematics while maintaining assistive functionality [31,40,54,56]. Overall, the reviewed studies demonstrated that rehabilitation outcomes were influenced not only by torque generation capacity but also by sensing modalities, control strategies, biomechanical alignment, and human-robot interaction design.

## Discussion

### Bridging Engineering Innovation and Clinical Reality

Over the last 10 years, significant progress has been made in knee exoskeleton research, particularly in actuator design, sensor integration, and control system development [38,39,48,54,56]. From an engineering perspective, the domain has evolved significantly in complexity. Nevertheless, a closer look at the literature reveals that clinical validation has not kept up with this advancement. The risk-of-bias assessment indicates that current evidence supporting knee exoskeletons is methodologically heterogeneous. Although engineering reporting and technical characterization are generally robust, clinical evidence remains constrained by small sample sizes and the moderate risk of bias. Therefore, the strength and generalizability of the current evidence regarding long-term clinical efficacy should be interpreted with caution.

Many published studies continue to be conducted in laboratory settings, primarily involving healthy subjects rather than those with neurological or orthopedic conditions [29,32,45,48-50,55]. Although these investigations are essential for confirming feasibility and safety, they fail to fully address the intricacies of rehabilitation in practice.

Motor-driven exoskeletons remain the leading technology in this area due to their ability to accurately adjust assistive torque and their effective integration with control frameworks that use torque and impedance principles [31,34,42,54,56]. In structured walking activities, these systems can consistently provide assistance that varies with the gait cycle phase. Nevertheless, achieving greater torque capability often requires larger actuators and transmission mechanisms, thereby increasing distal mass and inertia [31,40,42,54]. These mechanical additions may influence natural gait mechanics and metabolic demand, yet these effects are not consistently quantified. In rehabilitation settings, the trade-off between torque capacity and wearability becomes particularly relevant, as long-term use depends on comfort and tolerance rather than peak output.

Passive and hybrid systems have emerged as attempts to address these limitations by emphasizing transparency and reduced mechanical burden [32,40,49,52]. Passive elastic devices have demonstrated measurable biomechanical effects during controlled tasks such as squatting and jumping [49,52]. However, their assistance profiles are inherently task-specific and less adaptable to the variability observed in pathological gait. Hybrid rigid-soft architectures seek to preserve torque capability while improving interface compliance and load distribution [31,54,56]. Although conceptually promising, comparative data demonstrating superior long-term outcomes are still limited. Taken together, the current evidence suggests that future progress may depend less on maximizing mechanical output and more on refining the balance between assistance magnitude, comfort, and adaptability to individual impairment.

The review demonstrated that rehabilitation outcomes were strongly influenced by engineering characteristics such as actuation type, control architecture, and interface compliance, highlighting the importance of biomechanically aligned and task-specific exoskeleton design.

### Knee Biomechanics

The observed results in clinical and biomechanical parameters can be directly interpreted using fundamental knee biomechanics. When walking on a flat surface, knee biomechanics are characterized by coordinated changes in joint kinematics and kinetics, particularly during the initial stance phase, in which knee flexion and extension moments regulate shock absorption and stability [66,67]. During daily activities, tibiofemoral forces can reach 2‐4 times body weight during level walking and up to 5 times body weight during stair descent, highlighting the substantial mechanical demands placed on the joint [68,69]. These biomechanical requirements emphasize the need to develop an exoskeleton adaptable to various tasks.

**Figure 8.**
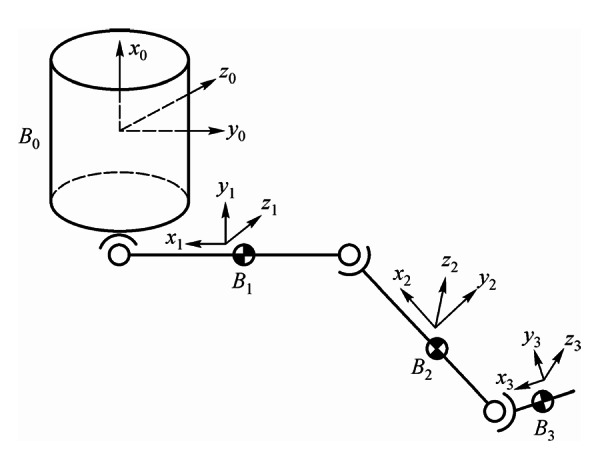
Mechanical model of human lower limbs. The figure illustrates the mechanical representation of the human lower limb modeled as a kinematic chain of rigid segments (pelvis, thigh, leg, and foot), each assigned a local coordinate system to define joint orientations and interactions [68].

Figure 8 shows a biomechanical model of the human lower limb, representing the femur and tibia as rigid bodies interconnected by ligamentous and contact elements, used to estimate knee joint kinematics and loading. This framework provides a physiological basis for designing knee exoskeletons that deliver assistive torques aligned with natural joint motion and internal force distributions.

Clinical data show that altered knee mechanics lead to pain and functional disorders. Although the peak moment of external knee adduction shows a weak overall correlation with knee pain in osteoarthritis, dynamic anomalies, such as varus deformity, are associated with pain, underscoring the importance of frontal-plane stability [70]. After total knee replacement, knee extension moments decrease in patients, and joint load increases during downhill walking, indicating continued biomechanical vulnerability in the early stages of rehabilitation [71]. Similarly, patients after anterior cruciate ligament reconstruction exhibit altered kinematics during rehabilitation [72]. Overall, knee exoskeletons must integrate accurate anatomical sensing, adaptive control, and compliant actuation to modulate joint moments without disrupting natural movement, underscoring the critical need to align engineering design with clinical biomechanics.

### Human-Robot Interface: A Determinant of Clinical Viability

Across studies, the quality of the human-robot interface consistently influences system performance, even when not explicitly analyzed. Mechanical misalignment between the anatomical knee axis and the exoskeleton joint can introduce parasitic torques and increase soft tissue stress [40,42,43,51,54]. Several groups have proposed self-aligning mechanisms, polycentric linkages, and improved suspension strategies to address this issue [33,42,54]. These refinements have been associated with improved user comfort and perceived naturalness of movement.

Despite these advances, many clinical investigations do not systematically report alignment errors, device migration, or long-duration wear tolerance [31,44,52,57]. This omission is clinically meaningful. Rehabilitation depends on repetition and sustained engagement; even minor discomfort or instability may alter neuromuscular activation patterns or reduce adherence over time. Emerging work examining embodiment and user perception suggests that when assistance feels mechanically coherent with voluntary movement, users integrate the device more effectively [44,57]. For this reason, interface optimization should be considered central to rehabilitation efficacy rather than an accessory design feature.

### Control Strategies: Reliability vs Personalization

Control architecture remains a defining feature of knee exoskeleton systems. FSM-based controllers remain widely used in clinical contexts for their deterministic phase segmentation and predictable assistance timing [21,31,33,38,39,41,45-47,51]. In patient populations where safety is paramount, this reliability provides practical advantages.

More advanced strategies, including impedance control [38,54], model predictive control [39], human-in-the-loop optimization [30], EMG-driven assistance [36,50], and neuromusculoskeletal model-informed machine learning [48], have demonstrated promising personalization capabilities in controlled environments. For example, human-in-the-loop optimization has reduced metabolic costs in healthy participants [30], and EMG-based systems enable modulation of assistance based on muscle activation signals [36,50]. However, these approaches are rarely evaluated in larger or clinically heterogeneous cohorts [44,57].

Furthermore, few studies report performance under fatigue, uneven terrain, prolonged daily use, or sensor drift [30,39,48]. Without such validation, it is difficult to determine whether algorithmic complexity yields meaningful clinical benefit. At present, clinical implementation appears to favor robustness and predictability over adaptive sophistication.

### Assistance Timing and Implications for Motor Learning

Across the reviewed literature, phase-specific assistance consistently contributes to functional improvement. Systems that target swing-phase knee flexion or stance-phase extension tend to improve toe clearance, knee extension, and gait symmetry while preserving voluntary muscle contribution [46,50,51,53]. This observation suggests that the timing of assistance may be more critical than continuous torque magnitude.

In contrast, trajectory-enforced systems may risk reducing active neuromuscular participation [42,44]. Since rehabilitation aims to facilitate motor relearning rather than to replicate passive movements, this distinction is important. Studies in pediatric cerebral palsy populations demonstrate that appropriately timed extension assistance can reduce crouch gait severity without suppressing voluntary engagement [50,51], supporting the concept of deficit-targeted assistance.

However, most outcome assessments are performed during active device use [31,44,57]. Postintervention follow-up is uncommon, making it difficult to determine whether improvements represent true motor adaptation or temporary mechanical augmentation. Longitudinal retention studies remain necessary to clarify this distinction.

### Clinical Interpretation and Functional Relevance

Short-term improvements in knee kinematics, gait symmetry, and metabolic efficiency were consistently reported across studies. However, these findings were predominantly derived from studies conducted in healthy participants under controlled laboratory conditions [30,31,40,49].

Evidence from clinical populations, including individuals with stroke, spinal cord injury, and cerebral palsy, remains comparatively limited and heterogeneous [29,42,44,51,57]. While some studies reported improvements in gait parameters and walking efficiency, these findings are based on small sample sizes, short-duration interventions, and variable study designs. Therefore, the strength of evidence supporting clinical effectiveness is substantially lower than that observed in healthy populations.

Importantly, most reported improvements occurred during active exoskeleton use, with limited assessment of retention or transfer of gains after device removal [31,44,57]. This raises uncertainty about whether the observed improvement represents true motor rehabilitation or temporary performance augmentation driven by mechanical assistance.

Short-term improvements in knee extension, step symmetry, and metabolic efficiency have been reported in stroke, spinal cord injury, pediatric cerebral palsy, and postsurgical populations [28,31,36,38,42,44,46,50,51]. In spinal cord injury, powered assistance reduced ambulatory effort compared to orthotic support [42]. Early postoperative implementation after total knee arthroplasty has been reported to be feasible and safe [28].

Nevertheless, most studies involve small cohorts and brief intervention periods [29,44,57]. Outcome measures frequently emphasize kinematic and biomechanical metrics, whereas participation-level outcomes and quality-of-life indicators are less consistently reported [35,37,53]. While biomechanical improvements confirm device functionality, sustained functional independence and integration into daily activities remain the ultimate rehabilitation goals.

The available evidence therefore supports short-term performance enhancement, but durable rehabilitation benefit has not yet been conclusively demonstrated. Larger randomized trials with extended follow-up are required to determine whether knee exoskeleton–assisted training yields meaningful, lasting functional gains.

### State of the Field and Translational Challenges

Collectively, the literature indicates that knee exoskeleton technology has achieved a high degree of mechanical and algorithmic sophistication [39,48,54,56]. However, clinical validation remains comparatively early in development [31,44,57]. The field appears technologically advanced yet still consolidating in terms of rehabilitation evidence.

Future progress will likely depend on closer integration between biomechanical analysis, interface refinement, control robustness, and longitudinal clinical evaluation. Demonstrating sustainable functional outcomes in real-world contexts will ultimately determine whether knee exoskeletons transition from promising prototypes to established rehabilitation tools.

### Study Limitations

This review is limited by substantial heterogeneity in device design, control strategies, populations, and outcome measures, which precluded quantitative meta-analysis. Many included studies were exploratory or feasibility-focused and frequently involved healthy participants, limiting generalizability to clinical populations. Reporting of mechanical specifications, adverse events, and long-term follow-up was inconsistent, constraining cross-study comparison. Additionally, most studies assessed outcomes only during device use, providing limited insight into retention or neuroplastic effects.

Furthermore, a substantial proportion of included studies were conducted in healthy participants or involved early-stage feasibility or prototype validation, limiting the direct clinical applicability of the findings.

### Future Research Directions

Future research should more explicitly align mechanical design and control strategies with specific rehabilitation objectives. Mechanical parameters such as torque capacity, range of motion, and assistance timing should be justified based on task demands and population needs rather than maximized by default. Adaptive and uncertainty-aware control approaches that show promise in healthy users should be systematically evaluated in clinical populations with attention to safety, usability, and interindividual variability.

Most importantly, future studies should move beyond short-term feasibility testing and controlled laboratory demonstrations. Well-designed, multicenter randomized controlled trials are needed, with larger, clinically representative samples, predefined primary outcomes, and standardized intervention protocols. Longer follow-up periods should be included to determine whether improvements persist after device removal and whether observed gains reflect true rehabilitation effects rather than temporary device-assisted performance.

Future trials should include patient-important outcomes, not only biomechanical parameters. These should include functional independence, walking, endurance, fall risk, participation in daily activities, quality of life, and user satisfaction. Greater standardization of outcome measures that integrate biomechanical, functional, and participation-level domains would strengthen cross-study comparisons and improve clinical interpretation. This would help determine whether knee exoskeletons can provide meaningful rehabilitation benefits beyond short-term improvements observed during assisted use.

### Conclusions

In summary, knee exoskeleton devices demonstrated consistent short-term improvements in biomechanical and performance-related outcomes, particularly under controlled laboratory conditions [30,31,40,49]. However, the current evidence is largely derived from small-scale studies, frequently involving healthy participants or short-duration feasibility trials [29,44,57].

Evidence from clinical populations remains limited and heterogeneous, with insufficient high-quality data to support routine clinical implementation in physical therapy and rehabilitation practice. While preliminary findings suggest potential benefits for gait rehabilitation, these results should be interpreted cautiously due to methodological limitations, including small sample sizes, lack of long-term follow-up, and variability in outcome measures [29,42,44,57].

Future research should prioritize well-designed, multicenter randomized controlled trials with clearly defined primary outcomes, longer intervention and follow-up periods, and inclusion of patient-centered outcomes such as functional independence, fall risk, and participation. These factors are critical for establishing the true clinical value and translational potential of knee exoskeleton-assisted rehabilitation.

## Supplementary material

10.2196/94916 Multimedia Appendix 1 Search strategy.

10.2196/94916 Multimedia Appendix 2 Filtering prompt and classification code.

10.2196/94916 Checklist 1 PRISMA 2020 checklist.

## Abbreviations:

EMG: electromyography
FSM: finite-state machine
IMU: inertial measurement unit
PRISMA: Preferred Reporting Items for Systematic Reviews and Meta-Analyses
PROSPERO: International Prospective Register of Systematic Reviews
RoB-2: Cochrane Risk of Bias 2
ROBINS-I: Risk of Bias in Nonrandomized Studies of Interventions
TKA: total knee arthroplasty
