# Supplementary material for: State-of-the-Art Knee Exoskeleton for Gait Assistance and Rehabilitation: Systematic Review
Source: JMIR Rehabil Assist Technol. 2026 Aug 4;13:e94916. doi: 10.2196/94916 (PMC13435957; doi:10.2196/94916)
Supplement: Supplementary file 2 [file rehab-v13-e94916-s001.docx]

**Search Strategy**

| **Databases** | **Search Strategy** | **Filters Applied** | **Date of Last search** |
| --- | --- | --- | --- |
| PubMed | (exoskeleton*[Title/Abstract] OR "robotic exoskeleton*"[Title/Abstract] OR "wearable robot* "[Title/Abstract] OR "powered orthos*"[Title/Abstract] OR "robotic orthos*"[Title/Abstract]) AND (knee [Title/Abstract] OR "knee joint"[Title/Abstract]) AND (human*[Title/Abstract] OR participant*[Title/Abstract] OR patient*[Title/Abstract] OR clinical[Title/Abstract] OR trial[Title/Abstract] OR experiment*[Title/Abstract]) NOT (simulation[Title/Abstract] OR simulated[Title/Abstract] OR "musculoskeletal model*"[Title/Abstract] OR "finite element*"[Title/Abstract] OR "computer model*"[Title/Abstract] OR "in silico"[Title/Abstract]) | English Language; Publication Data March 2025 | March 2025 |
| Scopus | TITLE-ABS-KEY (exoskeleton* OR "robotic exoskeleton*" OR "wearable robot*" OR "powered orthos*") AND (knee OR "knee joint") AND (human* OR participant*OR patient* OR clinical OR trial*) AND NOT (simulation OR simulated OR "musculoskeletal model*" OR "finite element*" OR "computer model*") | English Language; Publication Data March 2025 | March 2025 |
| Web of Science | TS= (exoskeleton* OR "robotic exoskeleton*" OR "wearable robot*" OR "powered orthos*") AND TS= (knee OR "knee joint") AND TS= (human*OR patient* OR clinical OR trial*) NOT TS= (simulation*OR "musculoskeletal model*" OR "finite element*" OR "computer model*") | English Language; Publication Data March 2025 | March 2025 |
| IEEE Xplore | Keyword phrase searches (“knee exoskeleton”, “robotic knee orthosis”, “wearable knee robot”); human testing and exclusion of simulation‑only studies assessed during screening | English Language; Publication Data March 2025 | March 2025 |
